# Iatrogenic Biliary Injuries: Multidisciplinary Management in a Major Tertiary Referral Center

**DOI:** 10.1155/2014/575136

**Published:** 2014-11-10

**Authors:** Ibrahim Abdelkader Salama, Hany Abdelmeged Shoreem, Sherif Mohamed Saleh, Osama Hegazy, Mohamed Housseni, Mohamed Abbasy, Gamal Badra, Tarek Ibrahim

**Affiliations:** ^1^Department of Hepatobiliary Surgery, National Liver Institute, Menophyia University, Shiben Elkom, Egypt; ^2^Department of Radiology, National Liver Institute, Menophyia University, Shiben Elkom, Egypt; ^3^Department of Hepatology, National Liver Institute, Menophyia University, Shiben Elkom, Egypt

## Abstract

*Background*. Iatrogenic biliary injuries are considered as the most serious complications during cholecystectomy. Better outcomes of such injuries have been shown in cases managed in a specialized center. *Objective*. To evaluate biliary injuries management in major referral hepatobiliary center. *Patients & Methods*. Four hundred seventy-two consecutive patients with postcholecystectomy biliary injuries were managed with multidisciplinary team (hepatobiliary surgeon, gastroenterologist, and radiologist) at major Hepatobiliary Center in Egypt over 10-year period using endoscopy in 232 patients, percutaneous techniques in 42 patients, and surgery in 198 patients. *Results*. Endoscopy was very successful initial treatment of 232 patients (49%) with mild/moderate biliary leakage (68%) and biliary stricture (47%) with increased success by addition of percutaneous (Rendezvous technique) in 18 patients (3.8%). However, surgery was needed in 198 patients (42%) for major duct transection, ligation, major leakage, and massive stricture. Surgery was urgent in 62 patients and elective in 136 patients. Hepaticojejunostomy was done in most of cases with transanastomotic stents. There was one mortality after surgery due to biliary sepsis and postoperative stricture in 3 cases (1.5%) treated with percutaneous dilation and stenting. *Conclusion*. Management of biliary injuries was much better with multidisciplinary care team with initial minimal invasive technique to major surgery in major complex injury encouraging early referral to highly specialized hepatobiliary center.

## 1. Introduction

Iatrogenic biliary injuries during cholecystectomy are a serious surgical complication that can have devastating consequences, including a significant risk of early death [1, 2].

Iatrogenic biliary injuries are feared complications reported to occur in approximately 0.2-0.3% in open cholecystectomy Era, but with incidence figures increasing following the introduction of laparoscopic cholecystectomy, with a mean figure of bile duct injuries when including both minor and major injuries up to 0.9% [3, 4]; this is initially attributed to a “learning curve phenomenon” which frequently occurs after introduction of any new procedure or technology [5].

Approximately 17–20% of biliary injuries were recognized intraoperatively [6].

The long-term implications for the patient, surgeon, and healthcare system along with the rising cost of litigation continue to mitigate this otherwise excellent procedure [7].

Traditionally, surgery has been the gold standard for the management of biliary injuries. Recently, various endoscopic and radiological intervention methods have been used as the preferred modalities of these patients [8], as they permitted a less invasive approach with similar or reduced morbidity rates at surgical treatment [9].

The management outcome of iatrogenic biliary injuries when it occurs has been shown to be better when such injuries are managed at specialized hepatobiliary center equipped with multidisciplinary service [10, 11].

The availability of surgical expertise to repair small caliber bile ducts high within the porta-hepatis and the availability of specialized radiological and endoscopic support are the main factors that contribute to the better outcome [12].

The choice of surgical reconstruction and timing of surgical repair are decisive for long-term course. Numerous surgical and interventional treatment modalities that are available require close interdisciplinary cooperation of gastroenterologists, radiologists, and surgeons [13, 14].

In this setting, we analysed the multidisciplinary management approach of iatrogenic bile duct injuries following cholecystectomy with emphasis on the improvement of long-term outcome in a major hepatobiliary referral center.

## 2. Patients and Methods

This retrospective study included 472 patients with iatrogenic bile duct injuries following cholecystectomy (open and Laparoscopic) referred to the Department of Hepatobiliary Surgery at National Liver Institute, Menophyia University, Egypt (a major tertiary referral center in delta region) from January 2002 to January 2012 and treated by multidisciplinary approach team including hepatobiliary surgeons, gastroenterologists, and interventional radiologists. The multidisciplinary team was established after ethical and scientific approval from Hepatobiliary Department and National Liver Institute committees. All cases of iatrogenic bile duct injuries should undergo this multidisciplinary team approach to set up a road map management of such cases.

All patients complained of postcholecystectomy biliary tract injuries encountered with variable presentation and timing from the surgical insult until they were referred to our center for further evaluation and management.

Cases were subjected to the following:

 thorough detailed history taking;
 meticulous clinical examination.

Operative details of the previous cholecystectomy should be revised with surgical team of referring hospital.

Investigation needed to diagnose the problems such as liver function tests and abdominal ultrasound were done for all cases as routine preliminary workup.

Computed tomography or magnetic resonance imaging was done in some cases.

Cholangiogram was done for all cases (the gold standard evaluation of biliary injuries) as a trans-tube cholangiogram (with a T-tube in place), an endoscopic cholangiography endoscopic retrograde cholangiopancreatography (ERCP) in most cases, or percutaneous transhepatic cholangiogram in some selected cases in which endoscopic approaches failed.

After receiving patients data by multidisciplinary team, patient condition was categorized through discussion of detailed results of treatment for each category to reach consensus on which type of modality to start with, either endoscopy or intervention radiology as minimal techniques for definitive treatment or bridging technique for definitive surgery (as complementary tool) prior to surgery or whether surgery still is needed for definitive treatment or surgery is mandatory from the start as definitive treatment.

Also the multidisciplinary team approach gave an outreach service for on-table repair of iatrogenic bile duct injuries to nearby hospitals around the tertiary center in 19 cases after receiving emergency call from the surgical team in those hospitals.

Patients were categorized according to the presentation into the biliary leakage group and the biliary stricture group as diagnosed by previous tools. Each group was managed according to the road map made by multidisciplinary team, starting with the minimally invasive tools (endoscopic treatment alone or in addition to percutaneous interventional radiological manipulation in difficult cases) to more invasive surgical treatment.

Biliary leakage group classified according to the classification of Strasberg et al. [15] was managed by endoscopic sphincterotomy in mild cases and/or stenting in moderate to major leakage, with concomitant stone extraction if present with the common bile duct (CBD) by ERCP.

Biliary stricture group categorized according to the classification of Strasberg et al. [15] was treated initially by endoscopic dilatation and stenting in repeated endoscopic sessions, with upgrading of the stent, until cure was obtained (after full dilatation of the stricture segment as evident by loss of the waist in the cholangiogram).

Percutaneous manipulation was attempted in cases of proximal biliary injuries as in major CBD injuries, transaction, or ligation through percutaneous transhepatic cholangiogram as diagnostic tool prior to surgery, percutaneous manipulations, and guide wire deployment through the CBD prior to combined procedures (Rendezvous) techniques or percutaneous dilatation and stenting for stricture or injuries.

Surgical approaches: surgical intervention was attempted for the cases not fixed by endoscopy or interventional radiology or cases which deserved surgical intervention from the start (transection, ligation, fibrotic stricture of CBD, and postoperative stenotic stricture in bilioenteric anastomosis (redo operation)), with the following surgical maneuvers:

- emergency surgery for peritoneal lavage and drainage of biliary peritonitis;
- on-table repair of iatrogenic bile duct injuries in cases diagnosed intraoperatively in our center or as an outreach service in nearby hospitals;
- primary repair on T-tube splint in a minor laceration injury of the CBD;
- choldocholithotomy procedure in associated CBD stones;
- undoing CBD ligation;
- bilioenteric anastomosis operations were done as a Roux-En-Y loop depending upon the site of injury, in proximal injuries in porta hepatis (Hepp-Couinaud technique), was capitalized on the extrahepatic course of the left main hepatic duct. Hepaticojejunostomy was done (for the injuries above the biliary confluence) in which the repair was done in the common hepatic duct or at the bile duct confluence with widening the stoma by opening the right and left bile ducts together at site of confluence (stomaplasty), or cholodochojejunostomy was done (in the injuries below the cystic duct insertion and the proximal bile and hepatic duct was not cicatrized or infected). The bilioenteric anastomosis may be side to side or end to side maneuvers depending upon the site and extent of the biliary injuries, and the anastomosis was tension free, mucosa to mucosa, and good wide stoma, with T-tube or biliary splint (specially small ducts) in majority of the cases to decompress the biliary tree in the immediate post-operative period and to obtain postoperative, contrast studies.

## 3. Results

This study was conducted on 472 cases of postcholecystectomy biliary injuries. The mean age was (46.8 years), with a range of 19–71 years. Out of 472 cases there were 302 cases (64%) females and 170 cases (36%) were males. Biliary injuries cases were 265 (56%) after laparoscope and 207 (44%) after open approach, with most of the cases of the open approach occurring at the late 5 years of study as the learning curve for laparoscopic approach reaches its saturation state and many surgeons, especially the young ones, become master of the laparoscopic technique without gaining good training in the open approach. only 24 cases (5%) were originally operated on in our center and 19 cases (4%) were operated on in the nearby hospitals as part of outreach service program for biliary injuries after urgent consultation from surgical team of those hospitals. Cases presented to our center within a month after operation were considered as early referrals and they were 274 cases (58%) including outstretch service, but the cases presented postoperatively after one month were considered late referrals and they were 208 cases (42%).

Cholangiogram was the main line of the diagnosis in cases of biliary injuries and was done in most of our cases. Also cholangiogram was the method of the diagnosing intraoperatively 5 cases in our center and 19 cases of outreach service program as intraoperative cholangiogram was done for those patients during or after the completion of the repair.

Cholangiography methods were done by endoscopy (endoscopic retrograde cholangiopancreatography (ERCP)) for 346 patients (73.4%), percutaneous transhepatic cholangiogram (PTC) was done for 24 patients (5%), magnetic resonance cholangiopancreatography (MRCP) was done for 61 patients (13%), intraoperative cholangiogram was done in 24 cases (5%), and complementary tests, combination of all these tests, were done for 17 patients (3.6%).

CT scan and MRI of the abdomen were done in most of the cases to detect any abdominal collection.

According to the results of cholangiogram, the injuries can be classified into biliary leakage and stricture group (Table 1).

 Biliary leakage group includes 288 (61%).

Cholangiogram demonstrated the following injuries:

 minor leakage in 93 patients (19.7%);
 major leakage in 52 patients (11%);
 possible transaction of CBD in 17 patients (3.6%);
 leakage with CBD stone shadow in 69 patients (14.6%);
 leakage with CBD stricture in 20 patients (4.2%);
 undetected leakage by cholangiography in 37 patients (7.8%) that may be due to minor leakage from bile ductules or gall bladder bed;
 biliary stricture group includes 184 patients (39%).

Cholangiogram demonstrated the following injuries:

 possible CBD ligation in 31 patients (6.5%);
 stricture in CBD
  - high stricture in 26 patients (5.5%),
  - middle stricture in 68 patients (14.4%),
  - low stricture in 24 patients (5%),
 stricture and stone in 18 cases (3.8%);
 postoperative bilioenteric stoma stricture in 17 patients (3.7%).

Treatment was done by either endoscopic approach (ERCP) alone or in conjunction with percutaneous approach or percutaneous approach alone or surgical approach after failing of the endoscopic or percutaneous approach or surgery from the start according to patient condition assessed by multidisciplinary team.

### 3.1. Endoscopic Treatment of Biliary Injuries (232 Cases (49%))

**Figure 1:**
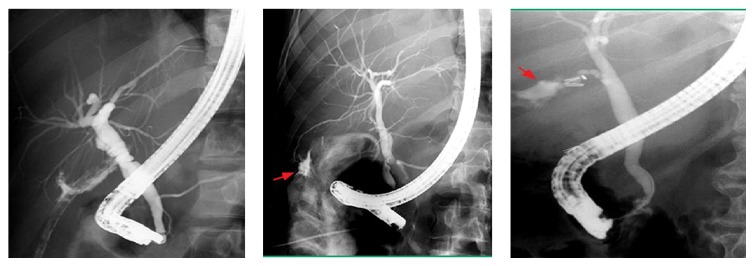
Endoscopic retrograde cholangiopancreatography showing minor biliary leakage from cystic duct stump and aberrant RHD radical, treated by sphincterotomy and stenting.

**Figure 2:**
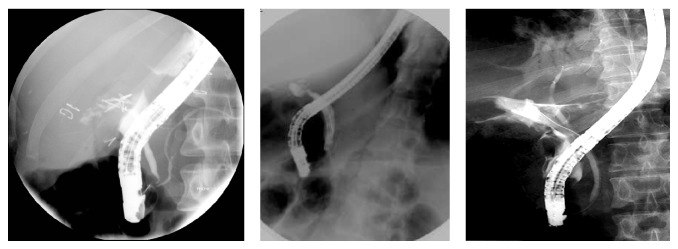
Endoscopic retrograde cholangiopancreatography showing a clipped, ligated common bile duct and a transection common bile duct with major biliary leakage.

**Figure 3:**
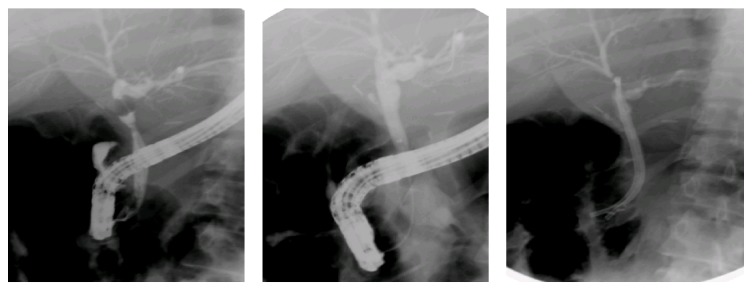
Endoscopic retrograde cholangiopancreatography showing common bile duct stricture treated by dilation and stenting.

Endoscopy was attempted in 232 patients (49%) using a side viewing videoscope, with regular instruments that were used in sphincterotomy and balloon dilatation and sphincteroplasty. Endoscopic treatments include sphincterotomy in mild cases and/or stenting in moderate to major biliary leakage, with concomitant stone extraction if present within the CBD (retrieval using basket, balloon extractor, or manual mechanical lithotripsy), and also dilatation and stenting in repeated endoscopic sessions with upgrading of stents until a cure was obtained (after full dilatation of the stricture segment as evident by loss of the waist in the repeated follow-up cholangiogram) (Table 2 and Figures 1, 2, and 3).

### 3.2. Percutaneous Manipulations Treatment of Biliary Injuries (42 Patients (9%))

**Figure 4:**
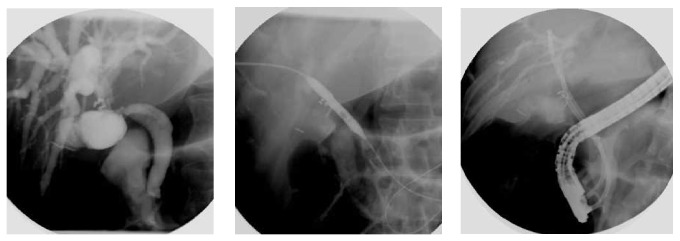
Rendezvous technique that followed PTC by dilatation and stenting of the CBD.

**Figure 5:**
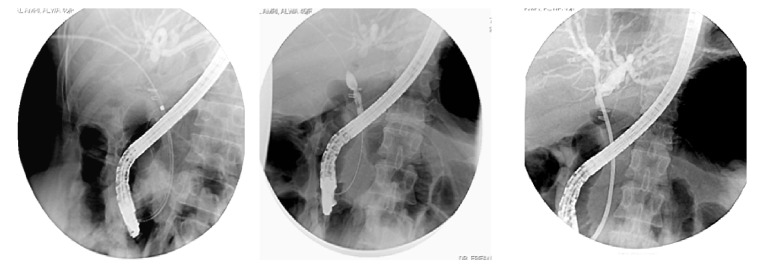
Rendezvous techniques with endoscopic stenting for common bile duct stricture.

**Figure 6:**
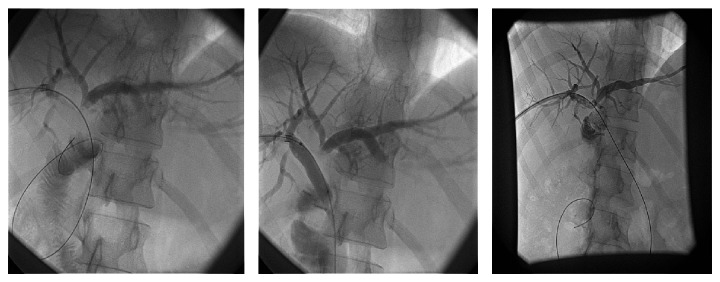
Percutaneous transhepatic dilation and stenting of the postoperative anastomotic stricture.

This approach was done in 42 patients after endoscopic failure in delineation of the proximal biliary tree as in the major CBD injuries, transection, or ligation through percutaneous transhepatic cholangiogram prior to surgery. Percutaneous manipulations and guide wire deployment through the CBD prior to combined procedures with conjunction with endoscopy (Rendezvous technique) in 18 patients or with other percutaneous techniques in the rest of the cases were attempted, where therapeutic dilatation and stenting for stricture or injuries were used in 14 cases and diagnostic PTC prior to surgery was used in the other 10 cases (Table 3 and Figures 4, 5, and 6).

### 3.3. Surgical Treatment of Biliary Injuries (198 Cases (42%))

Surgery was attempted in 198 cases (42%) either as an urgent surgery in 62 patients (including in-table repair in 19 patients in outreach service and 5 patients in our center) or as an elective surgery in 136 patients. In urgent surgery (62 patients) slipped cystic duct was ligated in 12 cases while peritoneal drainage and external biliary stents were inserted in 30 cases prior to further definitive treatment; however, it was a definitive treatment in 20 patients (17 patients in outreach service and 3 patients in our center).

**Figure 7:**
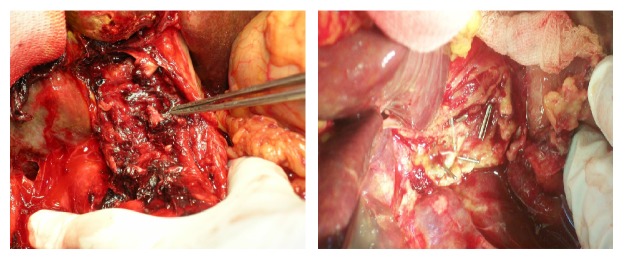
Operative photograph of ligated common bile duct with ligature (open) and clip (Laparoscopic).

**Figure 8:**
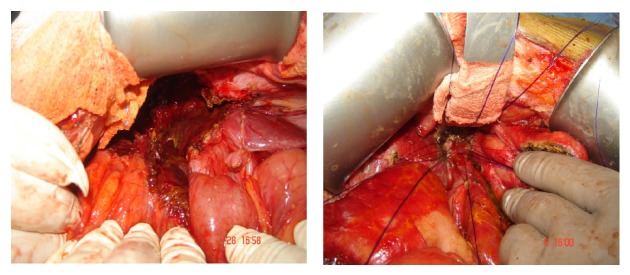
Operative photograph of meticulous dissection in porta hepatis to expose biliary injuries.

**Figure 9:**
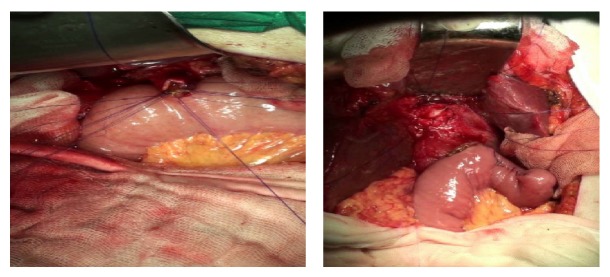
Operative dissections of hepatic ducts with Roux-en-Y loop hepaticojejunostomy anastomosis.

**Figure 10:**
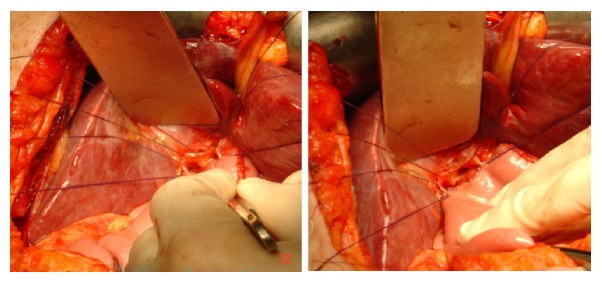
Operative hepaticojejunostomy and anastomosis of jejunum with single ostomy of both right and left hepatic ducts after operative stomaplasty.

The surgical maneuvers involved the following (Table 4 and Figures 7, 8, 9, and 10):

- peritoneal lavage and drainage for biliary peritonitis;
- drainage and ligation of slipped cystic duct ligature or clip;
- CBD repair on a T-tube splint in a minor lacerations injury in the CBD;
- choledocholithotomy procedure in associated CBD stones;
- undoing ligation and strictureplasty with a T-tube splint if CBD ligation is discovered early;
- bilioenteric anastomosis by Roux-en-Y hepaticojejunostomy.

### 3.4. Management and Follow-Up after Procedure

Routine postoperative management was carried out as follow. Endoscopically and percutaneously treated cases were regaining oral feeding 6 hours after the procedure and were discharged at the next day after the patient's condition became stable. Surgical cases were followed up in surgical ICU overnight and transferred to the surgical ward for a variable period prior to discharge (7–13 days). All cases were followed up for a period of 1.5–5 years after procedure.

### 3.5. Morbidity and Mortality

There was one (0.5%) mortality postsurgical maneuver due to biliary sepsis with secondary biliary cirrhosis due to long standing biliary stricture and obstruction. Complications were reported in each group of treatment optionally, postendoscopic maneuver complications were cholangitis, pancreatitis, and stent obstruction, and postpercutaneous manipulation complications were bleeding from PTC/PTD, biliary leakage around PTD, or slipped PTD catheter commonly reported; however, postsurgical complications were mainly wound infection, postoperative bile leakage in early postoperative period and postoperative intrahepatic stones, postoperative biliary stricture, and incisional hernia in the long-term follow-up (Table 5).

## 4. Discussion

Iatrogenic bile duct injuries pose a complex challenge to the treating physicians [16]. Simon wrote that “too many common bile ducts are still being cut during cholecystectomy” [17]. After decades of advent of laparoscopic cholecystectomy we still have too many common bile ducts injured during this operation. Obviously and luckily, bile duct injuries rate during cholecystectomy has fallen to more encouraging rate of 0.2% [18].

Inadequate management of bile duct injuries led to severe complications, such as biliary peritonitis leading to sepsis and multiple organ failure in early phase, and biliary cirrhosis during long-term follow-ups, and eventually the need for liver transplantation [19].

Not all forms of diagnostic workup and specialized treatments are available in all hospitals and there should be a low barrier for referral. Unfortunately, lesions will occur, but suboptimal treatment of biliary injuries is not accepted nowadays.

Our institute is a major referral center for hepatobiliary surgery with an increase in the flow of referral cases of postcholecystectomy biliary injuries. We adapted the multidisciplinary management approach program to deal with all cases of postcholecystectomy biliary injuries.

All cases of biliary injuries were reviewed by the multidisciplinary team following the steps of diagnosis and treatment.

In this series, all cases were subjected to a variety of diagnostic workups for diagnosis and delineation of biliary tract before any therapeutic intervention. In 7.8% of the cases diagnostic workup did not reveal any abnormalities which were considered as minor injuries and were treated conservatively without any intervention.

Management of biliary injuries detected during cholecystectomy is mainly dependent on the local expertise. If a competent hepatobiliary surgeon is not available, biliary drainage should be performed without exploration and patients should be referred to a highly specialized center as further exploration could lead to proximal extension of the lesion, sacrificing the normal healthy duct tissues, with having a negative impact on its reconstruction in the near future.

Multidisciplinary team has outreach service to nearby hospitals around our institute for immediate on-table repair of biliary injuries. In this series, 19 cases (4%) were treated as outreach service. In 17 cases, definitive treatment by repair of the bile duct over T-tube splint and hepaticojejunostomy anastomosis were performed with good long-term follow-up, while in other 2 cases biliary drainage was done for later further definitive treatment.

The advantages of immediate on-table repair of biliary injuries include single anesthesia, surgical procedure for the patient, and shorter hospital stay. When a hepatobiliary surgeon provides the service of on-table repair as an outreach service, in addition to the added advantage of better surgical outcome, the need to transfer the patient to a tertiary center is also abolished.

As opposed to a delayed repair, an immediate on-table repair nullifies the need for prolonged external biliary drainage and associated increases risk of sepsis. The disadvantages of such an outreach on-table repair of bile duct injures are that these injuries are often complex, requiring high hepaticojejunostomy reconstruction for nondilated, normal diameter (usually 3–8 mm) ducts with thin wall.

With our experience in living liver transplant at our center since 2003, our surgical team used to operate on normal bile ducts and becoming familiar with access to the site of injury was achieved satisfactorily since our outreach team brought a long suitable abdominal wall retractor and other instruments that are used for hepatobiliary surgery.

The extent of the ischaemic injury suffered by the bile duct is less apparent in the immediate repair setting [20, 21]. To reduce this, the proximal bile duct was divided up into a level where good blood from the cut surface of the duct occurred. This may explain why out of 17 cases of outreach service repair 2 cases developed late stricture of the hepaticojejunostomy requiring radiological dilatation.

The higher rate of injuries with laparoscopic method was initially attributed to the learning curve. This has, however, remained the same, a decade after the wide spread acceptance of the procedure [22, 23].

In this series, biliary injuries after laparoscopic approach were 56% of the total cases and 44% for open approach, with most of the cases of open approach occurring at the late 5 years of study as the learning curve for laparoscopic approach reaches its saturation state and many surgeons, especially the young ones, become masters of the laparoscopic technique without gaining good training in the open approach.

Bile leakage was a common presentation among our patients (61%), usually the leakage that originated from the liver bed or biliary injuries as documented by various studies [24], and can be explained also as the sphincter of Oddi creates a pressure gradient that results in bile spillage to outside rather than in the duodenum [25].

Bile leakage was demonstrated by cholangiogram in most of the cases (251 of 288 patients); however, the spillage was very mild and not evident by contrast injection in 37 cases (12.8%), such minimal bile leakage was resolved spontaneously which is concomitant to the stated facts in other literatures [26].

Endoscopic treatment in this series was applied in 232 (49%) cases of biliary leakage and stricture.

In this series, endoscopic treatment was achieved in mild and moderate cases up to 96–100%, as explained in the literatures that endoscopic treatment accelerates the healing period by decompressing the biliary system; in addition, it closes the defect physically and acts as a bridge at the site of extravasation. Stenting also acts as a mold and prevents stricture formations during recovery period and should be the preferred treatment [27].

In major leakage (type D&E) Strasberg classification endoscopic treatment with sphincterotomy and stenting was successful in 65% (34 out 52 cases) only. This result was compatible with other reports in [28–30].

Out of 34 cases 10 cases developed later stricture which was treated with upgrading the size of the stent; our results are also comparable with other literature reports [27].

Common bile duct stones were found to be exacerbating the bile leakage in 69 cases and were successfully treated by sphincterotomy and stone extraction in conjunction with stenting in 61 cases (88%) out of 69 cases. This result was in agreement with other reports [31, 32]. Also, common bile duct stricture found with leakage was treated by appropriate boogies or balloon dilatation and stenting in 8 cases out of 20 cases, in agreement with findings by other authors [33, 34].

In biliary strictures after biliary injuries the endoscopic treatment was successful in 67 patients with sphincterotomy, boogies or balloon dilatation, and convenient stenting. It was performed in conjunction with common bile duct stone extraction in 7 cases out of 18 cases and in repeated ERCP sessions to replace or subsequently upgrade the stent in 49 cases, in agreement with other previous reports stating that ERCP and stenting have good results with lower rates of morbidity and mortality [30, 33, 34].

Endoscopy is the preferable initial therapy in biliary leakage and stricture [35, 36], but it needs a long period (about 24 months) and repeated endoscopic sessions with progressive increase in the number of the stents to better calibrate the stricture [37].

Stents should be replaced every 3 months before possible clogging could cause cholangitis, and the patient should be informed about the risk of stenting and duration of the treatment [38–40].

Otherwise, surgery is indicated as the treatment of choice, especially in surgically suitable patient [37]. However, Davids and colleagues [35] reported equal relapse of 17% of both treatments.

Unfortunately, the role of endoscopy is weak in common bile duct transection injuries with leakage as only 4 cases out of 17 patients were endoscopically treated, in agreement with other studies demonstrating this low incidence of endoscopic treatment of such problems [30, 37].

Diagnostic percutaneous transhepatic cholangiography (PTC) was done in 10 cases prior to surgery in high proximal injuries not delineated by endoscopy and percutaneous transhepatic drainage (PTD) was inserted for 5 patients in bad condition for preoperative preparation for surgery in high ligation of common bile duct.

Stenting of stricture with leakage in high proximal injuries was done in 3 patients out of 20 patients. However, Rendezvous techniques plus endoscopy were performed in cases which failed endoscopy in 18 cases, in agreement with other reports in [41, 42].

Percutaneous dilatation and stenting for stenosis and stricture in post-bilioenteric repair was successfully performed in 6 cases out 17 cases with good long-term results, in agreement with several reports of the treatment of such postoperative biliary stricture at stoma side in bilioenteric anastomosis [41, 42].

Surgery was done in 198 cases (42%) of this series as an urgent surgery for the 62 cases, ligated slipped cystic duct (open or laparoscopic)was done in 12 cases, and peritoneal lavage with external biliary drainage was carried out in 30 cases; however, the surgery was definitive (on table-repair) in 20 cases and 13 cases were common bile duct repair over T-tube and in 7 cases bilioenteric anastomosis was done on-table repair as practiced by other authors [43, 44].

On other hand, surgery was needed as elective in 136 patients, especially after failure of other minimal invasive techniques (endoscopy and interventional radiology), and surgery was effective in common bile duct repair over a T-tube splint, choledocholithotomy, and common bile duct repair over T-tube splint, choledocholithotomy, strictureplasty and T-tube splint, and bilioenteric anastomosis, which was done in 96 cases as the operation of choice in most documented studies [45–47].

In this series, we used transanastomotic stents, the rationale that leaks of small bilioenteric anastomosis promote stricture and the rationale that both lowering of the intraductal pressure and adequate flow through the anastomosis were warranted by stents, as practiced by other authors [45, 48, 49].

Redo surgery in the post-bilioenteric anastomotic stricture or stenosis was done in 11 cases out of 17 cases with good long-term outcome.

The operation of choice in this series is Roux-en-Y hepaticojejunostomy as good long-term surgical results are obtained in this type of technique as documented in most literatures [45–47].

No mortality occurred in this series after endoscopic treatment, which is consistent with most reports in the literature [50]. But some minor complications were seen as cholangitis, pancreatitis, stent clogging, and bad patient compliance. Unfortunately, one death occurred following surgery (due to biliary sepsis leading to multiorgans failure) as well as some complications such as wound infection, bile leakage, incisional hernia and postanastomotic stenosis and strictures which were encountered in 3 cases only as our results are less than those of the reported in the literatures which state that stenosis occurs in about 10% of the cases after bilioenteric anastomosis [38, 45, 46, 48, 49].

All complication were treated conservatively except incisional hernia which was treated with hernia repair with mesh and postoperative anastomotic stricture, managed by percutaneous dilatation and stenting as it is very beneficial in such cases documented by other authors [41, 42].

## 5. Conclusion

The management of patients with biliary injuries should be ideally performed/discussed in a multidisciplinary team approach that consists of a gastroenterologist, radiologist, and surgeon. Better outcomes of such cases are mainly the result of multidisciplinary care and changes in technical aspects which have changed considerably through the time of learning curve with growing experience of the team. Early referral to high volume tertiary care center with experienced hepatobiliary surgeon, skilled gastroenterologist, and interventional radiologist would appear to be necessary to assure optimal results and should be encouraged.

## Figures and Tables

**Table 1:** Cholangiographic data.

Cholangiogram finding	*N*	%
Biliary leakage		
Minor leakage	93	19.7%
Major leakage	52	11%
Stricture		
High CBD stricture	26	5.5%
Middle CBD stricture	68	14.4%
Low CBD stricture	24	5%
Complex injuries		
Transection of CBD	17	3.7%
Ligated CBD	31	6.5%
Leakage and stone	69	14.6%
Leakage and stricture	20	4.2%
Stricture and stone	18	3.8%
Postoperative anastomotic stricture (stenosis)	17	3.7%
No abnormalities were detected	37	7.8%

Total	472	100%

CBD: common bile duct.

**Table 2:** Endoscopic treatment of biliary injuries.

	*N*	%
Endoscopic treatment		
Endoscopic sphincterotomy only for minor leakage	31	6.5%
Endoscopic sphincterotomy and stenting for mild leakage	47	10%
Endoscopic sphincterotomy and stenting for marked leakage	22	4.6%
Endoscopic sphincterotomy and stenting for transaction injuries	4	0.8%
Endoscopic sphincterotomy, stone extraction, and stenting for leakage with stones	51	10.8%
Endoscopic sphincterotomy with dilatation and stenting for leakage with stricture	8	1.7%
Endoscopic sphincterotomy and dilatation of ampullary stricture	13	2.8%
Endoscopic repeated dilatation with 8 French stents to 12 French stents		
Single stent (in CBD and CHD)	38	8%
Double stents (right and left hepatic ducts)	11	2.3%
Endoscopic dilatation of CBD stricture, stone extraction, and stenting for CBD stricture with stone	7	1.5%

Total	232	49%

CBD: common bile duct. CHD: common hepatic duct.

**Table 3:** Percutaneous radiological treatment of biliary injuries.

	*N*	%
Radiological treatment		
Diagnostic PTC prior to surgery for major CBD injuries	10	2.1%
PTC and stenting for stricture and leakage	3	0.6%
Rendezvous technique plus endoscopy for failed cases or stricture dilation and stenting	18	3.8%
PTD for ligated CBD in bad patient condition prior to surgery	5	1%
PTC and percutaneous dilatation and stenting for postoperative anastomotic stricture or stenosis	6	1.3%

Total	42	9%

PTC: percutaneous transhepatic cholangiogram. CBD: common bile duct. PTD: percutaneous transhepatic drainage.

**Table 4:** Surgical management of biliary injuries.

Surgical procedure	*N*	%
Urgent surgery (62 patients)		
Ligated slipped cystic duct (open or laparoscopic)	12	2.5%
Peritoneal lavage and external biliary stent	30	6.4%
CBD repair over T-tube in cases of injuries detected intraoperatively (on-table repair)	13	2.7%
Bilioenteric anastomosis in cases of injuries detected intraoperatively (on-table repair)	7	1.5%
Elective surgery (136 patients)		
Choledocholithotomy and CBD repair over T-tube splint	8	1.7%
Choledocholithotomy, strictureplasty, and T-tube splint	12	2.5%
CBD strictureplasty and repair over T-tube splint	9	2%
Bilioenteric anastomosis by Roux-en-Y hepaticojejunostomy (96 patients)		
Bismuth I injuries	40	8.5%
Bismuth II injures	31	6.6%
Bismuth III injuries (Hepp-Couinaud hepaticojejunostomy)	18	3.8%
Bismuth IV injuries with		
2-duct anastomosis with transanastomotic stent	4	0.8%
3-duct anastomosis with transanastomotic stent	3	0.6%
Redo surgery		
Repeated bilioenteric anastomosis for postoperative stricture and stenosis	11	2.3%

Total	198	42%

CBD: common bile duct.

**Table 5:** Morbidity and mortality.

Procedure	*N*	%
Endoscopic maneuvers (232 cases)	29	12.5%
Cholangitis	9	7.7%
Pancreatitis	3	2.6%
Stent occlusion	8	14.6%
Bad patient compliance	9	5.2%
Mortality	0	0
Percutaneous maneuvers (42 cases)	5	12%
Biliary leakage around the PTD	2	4.8%
Bleeding from PTC and PTD	1	2.4%
Slipped PTD catheter	2	4.8%
Mortality	0	0
Surgical procedures (198 cases)	22	11%
Postoperative bile leakage	7	3.5%
Wound infection	8	4.5%
Postoperative intrahepatic stones	2	1.5%
Postoperative biliary stricture and stenosis	3	1.5%
Incisional hernia	2	1%
Mortality	1	0.5%

PTD: percutaneous transhepatic drainage.
